# Coming Exodus of Trade

**Published:** 1875-01

**Authors:** Ben. H. Pratt


### THE BISTOURY.

Vol. X. ELMIRA, N. Y., JAN., 1875. No. 4.

### Contributions.

### THE COMING EXODUS OF TRADE.

BEN. H. PRATT.

The first men were producers by necessity. Agriculture was the only source of production. Daily bread, in the beginning, was the result of the toil and patience of the eater. Life was given man. The gift was a gratuitous one. It is a question with biologists whether the life so given was prized *per se*, or whether the parting with it was so dreaded that men chose the lesser evil, and preferred the toil that supported, rather than the idleness that was fatal to the life. At all events, there was but one condition upon which life could be retained. That condition was work. "By the sweat of thy brow thou shalt earn thy bread." Production, through the processes of agriculture, was the first, the essential, the only occupation by which men could fulfill the divine injunction. Hence all men were producers.

By and by the husbandman produced more than he could eat. Production was in excess of consumption. Idleness had not yet been recognized as even a tolerable state of existence. The fruits of the earth being the first objects upon which the human mind dwelt, and appetite stimulating desire for a higher order of sustenance, a developement of that which had been produced drew off from the number of producers, and the equilibrium was restored. Hence we come upon the origin of a second class of toilers, the developers. At this stage the developers and producers were the consumers. As the race of men spread more widely, and production and development embraced more workers than the needs of consumption required, the middle-men arose. These neither produced any thing nor developed that which had been produced. They merely handled. Transportation became necessary, at first by the more simple, then by more complex methods. This by a very natural process, revealed another necessity, places wherein to store the accumulations of the transporters. Buying and selling, or their equivalent, barter, became a recognized occupation, and the merchants, a new class, arose. For their accommonation, builders of rude receptacles for merchandise—bazaars, as it were—where men might gather for the purpose of procuring such articles as their necessities demanded, became a necessary adjunct of society. Inasmuch as it is a law of nature that men are never satisfied with what they have, but are continually demanding more and more, the gratification of the necessary wants is followed by a desire for the supernecessary, the luxurious. Here are introduced a long line of producers and developers. The minerals, so far as their existence was known, were sought after as necessity and luxury demanded. Artificers in all known material sprang up, skilled workmen being more and more in demand.

Without going further into the origin of the three great classes, producers, developers and middlemen, let us note the characteristics of each. The producer is a toiler. He cannot acquire without work. His labor is manual, fatiguing, exhaustive, wearing. So long as no other field of occupation presented itself, men were universally satisfied with this. The developer is also a toiler. He cannot manipulate the material brought to his hands by the producer without hard labor, but there is more thought and skill required. His work is less irksome, more intellectual, requires more ingenuity, is more attractive. Hence, even in the earlier periods, there was great temptation to enter the ranks of development rather than those of production. But when the middle-men arose, whose occupation was less a toil, and by reason of their association with men and the varied character of their daily life, bore with it a sort of fascination, then the temptations to enter this, rather than either of the other grand divisions of human employment, were great.

In this article, for a cause, we do not enter upon the fields of mental labor, so considered, at all. The professions, the higher ranks of art and literature, the brokers, the bankers, the men who live upon their wits, and the like, are regarded as outside the pale of manual labor entirely—a different sort of men. And right here we may as well tell what we are driving at, lest the reader, if we have one, may not perceive the drift or what has gone before.

We have too many middle-men—that is to say, too many merchants of one sort and another—men who live by handling the results ot the producers' and developers' labor. The causes of this are evident. As civilization advances the antipathy to manual labor becomes greater from generation to generation. The farmer's son, wearily returning from his father's fields at night-fall, forgets his independence, does not remember the honorable calling that enabled his father to maintain his manhood and pursue a straightforward course among his fellow men, fails to appreciate the blessings that rurality and a life in accordance with nature's laws have vouchsafed him. He ignores all save that his lot is one of daily toil, and he meditates, while on his couch at night, upon the fascinations of the town, the social intercourse with men, the comparative ease of the merchant's life. He would accept the meanest position in trade—prefer the office of a sweeper of floors, tender of fires, runner of errands, and perform the veriest drudgery of the shops where men buy and sell and bargain from sunrise to bedtime, rather than follow the plough or bend to the cradle during the hours of sunlight and in a few years succeed to the honors and responsibilities of a landed proprietor. The son of a manufacturer, dreading the busy hum and clatter of the works, and the association with dingy faces and oily clothes, begs to be allowed to turn his back upon these scenes and accept the most humble position in trade. Men who fail to accomplish the ends in life to which they once aspired, rush into the mercantile business pell mell, preferring even to brave the inclemency of the seasons, behind a peanut stall, than put their hands to toil. No other branch of industry seems to lure so effectually as that which the merchant controls. Hence the spheres of the producer and the developer are being continually deprived of their strength and numbers, while the ranks of those who handle, but do not produce or develop, are increased beyond all measure. Thus, trade is filled to overflowing, and the strife for business compels those engaged in it to resort to all manner of trick and device and over-reaching in order to gain patronage, while among the producers there is a great and unanswered outcry for help at certain seasons, and an annual waste of those products of the soil, that which are the bone and sinew of our national strength, is the result. In our shops and factories and mills, where the development of the agricultural and mineral wealth of our country is going on, there is a like unanswered outcry, though it be not for workers so much as for skilled work, for workmen whom education and a higher development of the intellectual might endow to the devising of modes of manufacture still more intelligent than those we now possess. But the sons of our wealthy men, having spent their youth in the cultivation of their mental powers, return from college with notions above the office or shop which provided the means for their education. Hence our labor-saving machinery and the processes by which the raw material is converted into the finished article, although wonderful almost beyond belief, are still rude compared with what they shall be when their construction shall become the result of the intellectual as well as the practical mind.

It has become not only an every-day remark that the mercantile business is over-crowded, and is robbing other pursuits of the men who might therein better serve themselves and their fellow men, but the necessity of doing something to drive the supernumeries out of trade is most keenly felt by those who take a deeper and a broader view of business economy. If some such step be not taken the matter must ere long right itself by the failure of a very large share of those in the mercantile line to make even a living. Even now the very low salaries that are paid to clerks, salaries that seem as wretchedly small compared with the wages of some of those engaged in the mechanical trades, are an evidence that clerkships are far fewer than the candidates for them. There are always a large number of young men on the alert for positions in a "store," so many, that they who did not know to the contrary would imagine that the position of a clerk is not only an enviable one but exceedingly remunerative. On the contrary, it is neither, for if there is a poorly paid slave in all the community, he is the ordinary clerk who smiles at you from behind the counter by day and groans out his hatred of his work when the day's toil is over. What peculiar fascinations linger about this business we cannot perceive, save those we have mentioned in the earlier part of this article, and these seem to us a very poor compensation for the ills that wait on the monotonous and confining duties of the genus clerk. It is a pity that our young men are above learning trades and thus becoming independent, in a great degree. It is a pity that parents discourage their children from pursuing the bent of their genius, which should always be consulted. This false pride which makes manual labor and mechanical trades vulgar, and the effeminate clerkships genteel, is doing a greater injury to business and society and the welfare of the nation than any other mistaken sentiment we recognize.

